# Cu-THQ-EFG Composite for Highly Selective Electrochemical CO_2_ Reduction to Formate at Low Overpotentials

**DOI:** 10.3390/polym14235112

**Published:** 2022-11-24

**Authors:** Lisha Jia, Klaudia Wagner, Jamie Smyth, David Officer, Jun Chen, Pawel Wagner

**Affiliations:** ARC Centre of Excellence for Electromaterials Science, Intelligent Polymer Research Institute, University of Wollongong, Wollongong, NSW 2522, Australia

**Keywords:** metal organic frameworks, electrochemical CO_2_ reduction, edge-functionalized graphene

## Abstract

Metal organic framework (MOFs) are promising materials for electrocatalysis. However, the active sites of bulk MOFs crystal normally cannot be fully utilized because of the slow reagent penetration of pores and blockage of active sites. Herein, we report a facile way to deposit copper-benzoquinoid (Cu-THQ) on the edge-functionalized graphene (EFG) which prevented material’s aggregation. EFG used as a substrate provides higher electrical conductivity and stability in water than previously utilized graphene oxide (GO). Besides, the plate-like morphology of EFG proved to be more beneficial to support the MOF, because of the functional groups on its edge regions and much lower resistance compared to the sheet GO. Therefore, EFG can boost the resultant material’s catalytic activity for CO_2_ electroreduction (CO_2_RR). Furthermore, Cu-THQ exhibits high selectivity for formate formation in CO_2_RR. Representing as the only CO_2_ reduced liquid product, formate can be separated from gaseous products and further extracted from the electrolyte for practical use. The electrocatalytic results of Cu-THQ-EFG indicate the composite exhibits a higher current density of −3 mA/cm^2^ and faradaic efficiency of −0.25 V vs. RHE, corresponding to 50 mV of overpotential. Moreover, it features a less negative on-set potential of −0.22 V vs. RHE, which is close to the equilibrium potential of CO_2_RR (−0.2 V vs. RHE) and is 0.16 V more positive than the on-set potential of Cu-THQ-GO (−0.38 V vs. RHE).

## 1. Introduction

Electrochemical CO_2_ reduction reaction (CO_2_RR) is a promising way to reach the goal of carbon neutrality on the Earth and has delivered abundant benefits to the atmospheric CO_2_ conversion in the past three decades [1]. CO_2_RR can be vividly described as the reverse process of the fuel cell [2]. To be specific, it converts electricity driven by the renewable energy generating from solar, wind and wave, into the chemical energy and concurrently reduce superfluous CO_2_ to value-added carbon-based products. The efficiency of the conversion of the electrical energy to chemical energy can be quantified by the overpotential, which is defined as the difference between the actual potential of the reaction and the thermodynamic equilibrium potential (TEP) [3]. CO_2_ is a linear central symmetric molecule and has two C=O bonds, which is chemically stable and requires high energy (~750 kJ mol^−1^), that is, high potentials in electrocatalysis, to dissociate the bond [4]. The meaning of involving catalysts is, take CO_2_-to-formate ($CO_{2}+2H^{+}+2e^{-}\to HCOOH$) for example, to drive the conversion at a potential as close to −0.2 V vs. RHE as possible [5,6]. Due to low selectivity of the catalysts, normally more than one gas or liquid products were produced during the electrolysis, which will cost more to purifying the targeted products. Necessarily, hydrogen evolution reaction (HER, $2H^{+}+2e^{-}\to H_{2}$) with a low TEP as −0.019 V vs. RHE happens readily and compete with CO_2_RR in aqueous electrolyte, resulting the decreased efficiency. In fact, researchers are still in the primary process of exploring the commercialized catalysts for CO_2_RR, which can be distinguished by its long-term operation, high current density and excellent FE% when employed in the industrial applications [7].

MOFs are organic-inorganic hybrids assembled from metal ions (or clusters) and organic ligands [8]. MOFs featuring porous structure with significantly high surface area (500–6240 m^2^ g^−1^) work well as desirable catalysts for mainly CO and formate production from CO_2_RR [9,10,11,12,13,14]. MOFs as heterogeneous electrocatalysts usually provide a porous network for the reticulation of catalytic molecular for CO_2_RR and maximize the number of active sites by controlling their morphology and thickness [15]. Cu-based MOFs acting as catalysts have demonstrated great potential for the production of formate [16]. However, those MOFs suffer from unsatisfying ion diffusion capacity, low catalytic sites utilization and low electrical conductivity [17]. The noticeable shortcoming can be addressed by growing MOF on the thin substrate, such as GO or EFG, which is capable of not only boosting the conductivity but also alleviating the aggregation of the bulk MOF. EFG is composed of a few stacks of graphene-like sheets grafted with functional groups on the defect sites located exclusively on the edges. This material was first reported by Baek and co-workers [18,19]. The plate-like morphology of EFG indicates it is appropriate to be utilized as the substrate and the functional groups: carboxyl and hydroxyl can play their role as anchors to connect the MOF crystals on the substrate. The high dispersibility of EFG in organic solvents facilitates MOF composites [20,21]. Herein, we report a facile approach to creating Cu-THQ MOF and EFG composite for efficient CO_2_ electroreduction at room temperature and ambient pressure. The EFG presence in the composite increases the active surface area, conductivity and stability of the electrode, further promote its electrocatalytic performance.

## 2. Materials and Methods

### 2.1. Material and Chemicals

Copper(II) nitrate trihydrate (Cu(NO_3_)_2_.3H_2_O, 99.0%), tetrahydroxy−1,4-benzoquione hydrate (THQ, 99%) and ethylenediamine ( NH_2_CH_2_CH_2_NH_2_, 99.5%) from Sigma-Aldrich ((Sydney, NSW, Australia) were used as obtained. Carbon cloth (CC, plain) was purchased from Fuel Cell Store (College Station, TX, USA) and Nafion (5 wt%) from Ion Power (New Castle, DE, USA) were used as flexible substrate and binder in working electrode fabrication process, respectively. Sodium bicarbonate (NaHCO_3_, 99.9%) from Chem-Supply (Gillman, SA, Australia) was used for electrolyte. GO was synthesized by Hammer method described in previous reports [22,23]. EFG is prepared following the literature [24,25].

### 2.2. Synthesis of Cu-THQ-EFG Composite

**Scheme 1:**
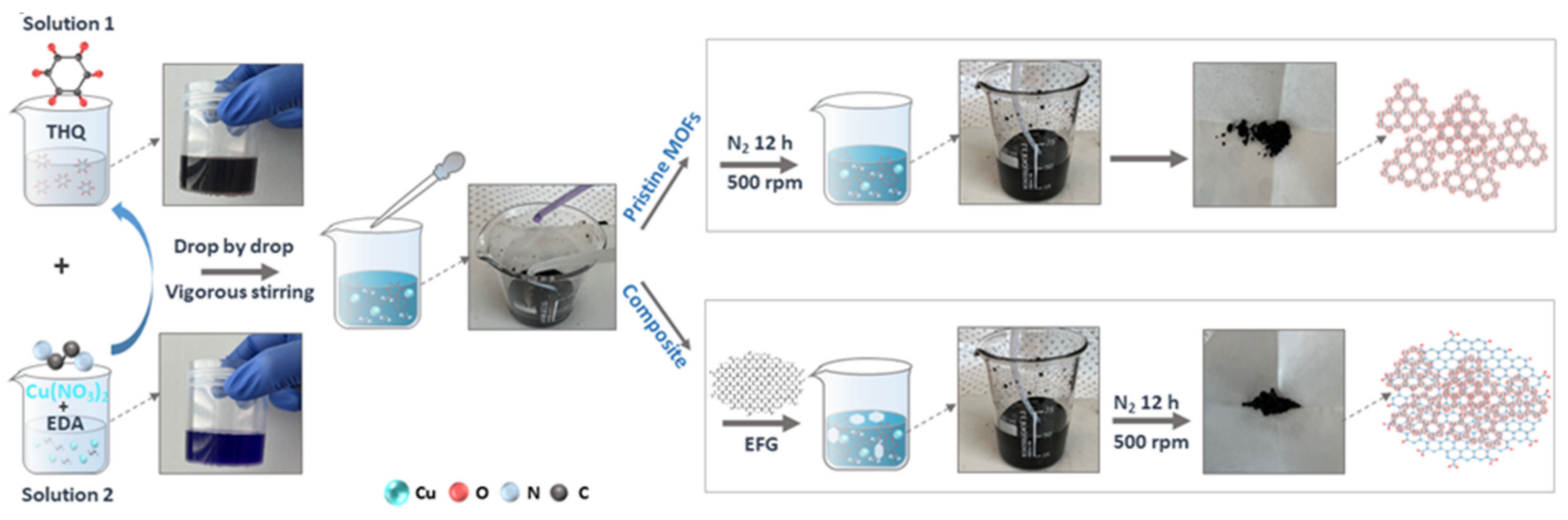
Schematic illustration of the fabrication process of the pristine Cu-THQ MOF and Cu-THQ-EFG composite.

Scheme 1 illustrates the synthesizing procedure of the pristine Cu-THQ MOF and Cu-TEQ-EFG composite. To be specific, firstly, 60 mg (0.45 mol) THQ ligand was dissolved in degassed DI water (20 mL) noted as solution 1; then 110 mg (0.45 mol) Cu(NO_3_).3H_2_O was dissolved in degassed DI water (20 mL) containing ethylenediamine (46 μL) noted as solution 2. The solution 2 was then slowly added in to the solution 1 drop by drop with N_2_ bubbling and vigorous stirring all the time. For Cu-THQ-EFG composite, after adding 11.6 mg EFG powder into the mixture of the solutions 1 and 2, the reaction continued to be stirred magnetically at 500 rpm for 12 h at ambient temperature, then the precipitate was separated and washed by centrifuging three times with DI water and three times with acetone. After drying at 60 °C for 2 h, the dark navy powder was obtained. For pristine MOF, the mixture solution was directly stirred magnetically at 500 rpm for 12 h at ambient temperature, following the steps in literature [26]. The molecular structure of MOF is shown in the Scheme 1. Cu-THQ-GO composite was also synthesized as the control sample under identical conditions and its procedure is the same as for Cu-THQ-EFG, except adding 1.5 mL GO (7.76 mg/mL) in the mixture of solutions 1 and 2.

### 2.3. Materials Characterizations

The X-ray powder diffraction (XRD) of MOF crystal was measured on PANalytical Empyrean with a Cu Kα radiation source (λ = 1.5406 Å) at a generator voltage of 45 KV and a generator current of 40 mA with a scanning speed of 3°/min. Raman spectroscopy was conducted on HORIBA scientific-LabRAM HR Evolution with an excitation wavelength of 633 nm and grating of 300 (600 nm). The X-Ray photoelectron Spectrometer System (XPS) from Thermo Scientific Nexsa Company (Madison, WI, USA) was used to scan the spectra of elements. The cluster etch mode of XPS was employed with ion energy of 5000 eV for 20 s. All the spectra were calibrated by C 1 s (284.8 eV). A scanning electron microscope (JSM-7500) at an operating voltage of 15 KV and a transmission electron microscope (JSM-F200) from JEOL Company (Frenchs Forest, NSW, Australia) were used to investigate the morphology of catalysts and the mapping images of all elements in the material. Thermo-gravimetric analysis (TGA) was measured on a TG 209 from NETZSCH Company (Selb, Germany) in air atmosphere with heating rate 3°/min to investigate the thermal properties of catalysts and the loading mass of the Cu-THQ in Cu-THQ-EFG composite. The N_2_ adsorption and desorption test of materials were conducted by Bruauer-Emmett-teller (BET) measurements using a TriStar II 3020 surface area and porosity analyzer from Micrometritics Company (Caringbah, NSW, Australia).

### 2.4. Electrocatalysis Characterizations

#### 2.4.1. System Set-Up

All the electrochemical measurements were tested on CHI 660 potentiostat from CH Instruments Company (Bee Cave, TX, USA) with the automatic iR compensation function, using three-electrode H-cell with two compartments separated by Nafion 117 membrane. Each compartment contained 30 mL 0.1 M NaHCO_3_ electrolyte. The 1 cm×1 cm carbon cloth sprayed with catalysts ink with loading mass 1 mg/cm^2^ was used as a working electrode and Ag/AgCl (3 M NaCl) as a reference electrode both installed in the cathodic compartment and the anodic compartment was set up with the 1 cm×2 cm platinum sheet as counter electrode. Ar and CO_2_ were bubbled through the cathodic compartment through the AALBORG mass flow controller and the cell was also linked with gas chromatography (GC) Shimadzu GC-2030 from SHIMADZU Company (Sydney, NSW, Australia) using a plastic tube to transfer produced mixture gas directly to GC FID and TCD columns for analysis. The whole system (Figure S1) was thoroughly gas tight.

#### 2.4.2. Electrochemical Measurements

To fabricate working electrode, 1 mg catalyst was mixed with 1 mL ethanol and 10 μL nafion solution (5 wt%), the mixture was sonicated for 1 h until uniform catalyst ink was formed. Then, the ink was sprayed by 1 mL plastic syringe fitted with a gauge needle (25G × 1”, 0.50×25 mm), on one piece of clean carbon cloth (1 cm × 1 cm) and used as working electrode with loading mass 1 mg/cm^2^ and 0.83 mg/cm^2^ for Cu-THQ and Cu-THQ-GO/EFG, respectively. All the potentials were measured against the reference electrode and converted to the reversible hydrogen electrode (RHE) reference scale by the following equation, $E_{RHE}=E_{Ag/AgCl}+0.21+0.0591\times pH$. The pH value of CO_2_ saturated 0.1 M NaHCO_3_ aqueous solution was 6.8. Prior to the measurements, the working electrode was in situ activated for 5 min at −1.2 V vs. Ag/AgCl with bubbling Ar. After activation, Ar was continued to flow in the cathodic electrolyte for another 25 min in order to remove the O_2_ from the electrolyte. Then, CO_2_ was bubbled into the electrolyte for 30 min to obtain a CO_2_ saturated stable system for the test. Linear sweep voltammetry (LSV) was tested in Ar and CO_2_ saturated electrolytes with 20 mV/s scan speed. Electrochemical impedance spectroscopy (EIS) was carried out in a CO_2_-saturated 0.1 M NaHCO_3_ solution at −0.25 V vs. RHE with an amplitude of 5 mV in the frequency range of 0.01 to 100 KHZ. Constant potential electrolysis (CPE) was carried out for 30 min for each fixed potentials, the produced gas product were online injected into GC instrument for product analysis.

#### 2.4.3. CO_2_ Reduction Product Analysis

Online GC was employed to analyze the gaseous product produced at the cathode. The catholyte was magnetically stirred at 500 rpm to enhance the mass transport of CO_2_. Every measurement proceeds at different potentials from −0.1 V to −0.6 V after catalytic reduction was carried out for 30 min and the gaseous product with the CO_2_ flowing was vented into the gas-sampling loop (1 mL) of the GC, which was equipped with a flame ionization detector (FID) and a thermal conductivity detector (TCD). Specifically, FID with methanizer was utilized for quantifying CO, CH_4_, C_2_H_4_ and C_2_H_6_ correspondingly. TCD was used to quantify H_2_ and CO_2_. The molar ratio of CH_4_, CO and H_2_ in the 1 mL analyzed gas can be calculated by using the calibration equation as shown in Figure S2a–c. Liquid product was analyzed by a 400 MHz Nuclear magnetic resonance spectroscopy (NMR) from Bruker Company (Sydney, NSW, Australia) at 28 °C. A 500 μL product-containing electrolyte was syringed out from the cathodic compartment after 30 min electrolysis. It was mixed with 100 μL of internal standard of 0.6 mM DSS (3-(trimethylsilyl)-1-propanesulfonic acid sodium salt, Sigma-Aldrich, >99.7%) and 100 μL of D_2_O (99.9%) from Cambridge Isotope Lab Company (Tewksbury, MA, USA). The 700 μL mixture solution was sonicated for 2 min and then transferred into a clean and dry NMR sample tube for further analysis. The relative area value of the formate peak at 8.44 ppm to the DSS peak at 0 ppm can be fitted, then based on the linear relation between concentration of formate and relative area of formate peak calibrated in Figure S2d, the molar concentration of formate in 30 mL of electrolyte can be calculated.

## 3. Results and Discussion

### 3.1. Structure Analysis

**Figure 1:**
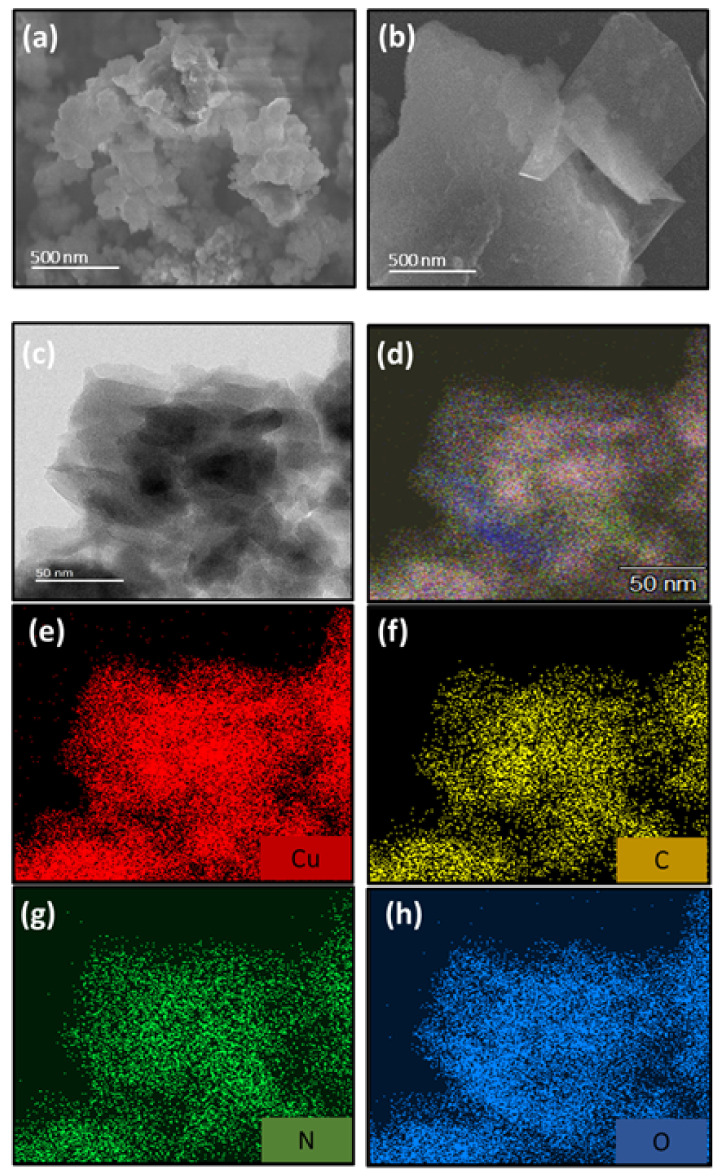
(**a**,**b**) SEM images of the Cu-THQ MOF and Cu-THQ-EFG, respectively; (**c**) TEM images of Cu-THQ-EFG; (**d**–**h**) the corresponding element mapping results of Cu, C, N and O in Cu-THQ-EFG.

SEM image of Figure 1a reveals the polycrystalline structure of Cu-THQ MOF. However, the bulk MOF suffers extensive aggregation with relatively large particle size of ~400 nm. Figure 1b shows layered plate-like morphology of Cu-THQ-EFG, which clearly exhibits the MOF is deposited on the surface of EFG, preventing the aggregation of MOF consequently, thereby increasing the exposure of active sites in the MOF. Besides the SEM image of Cu-THQ-GO in Figure S3 also presents layered sheet-like structure with less aggregation compared with the pristine Cu-THQ.

Additionally, TEM image of Cu-THQ-EFG in Figure 1c further confirmed the layer-by-layer morphology of composite assembled by the plate-like shape of EFG with the deposition of MOF and the carbon region of EFG can also be evidently observed in the TEM image. the corresponding energy dispersive spectroscopy (EDS) elemental mapping analysis, as shown in Figure 1d–h, identify the existence and uniform distribution of elemental Cu, C, N and O in the composite. The N comes from the ethylenediamine involved in the synthesis of MOF [27]. Figure S4b shows three different planes in the composite, which belong to (020), (002) and (110) of Cu-THQ MOF with d_020_ = 10.6 Å, d_002_ = 14.3 Å and d_110_ = 11.6 Å, respectively. It further proved the Cu-THQ phase in the composite.

**Figure 2:**
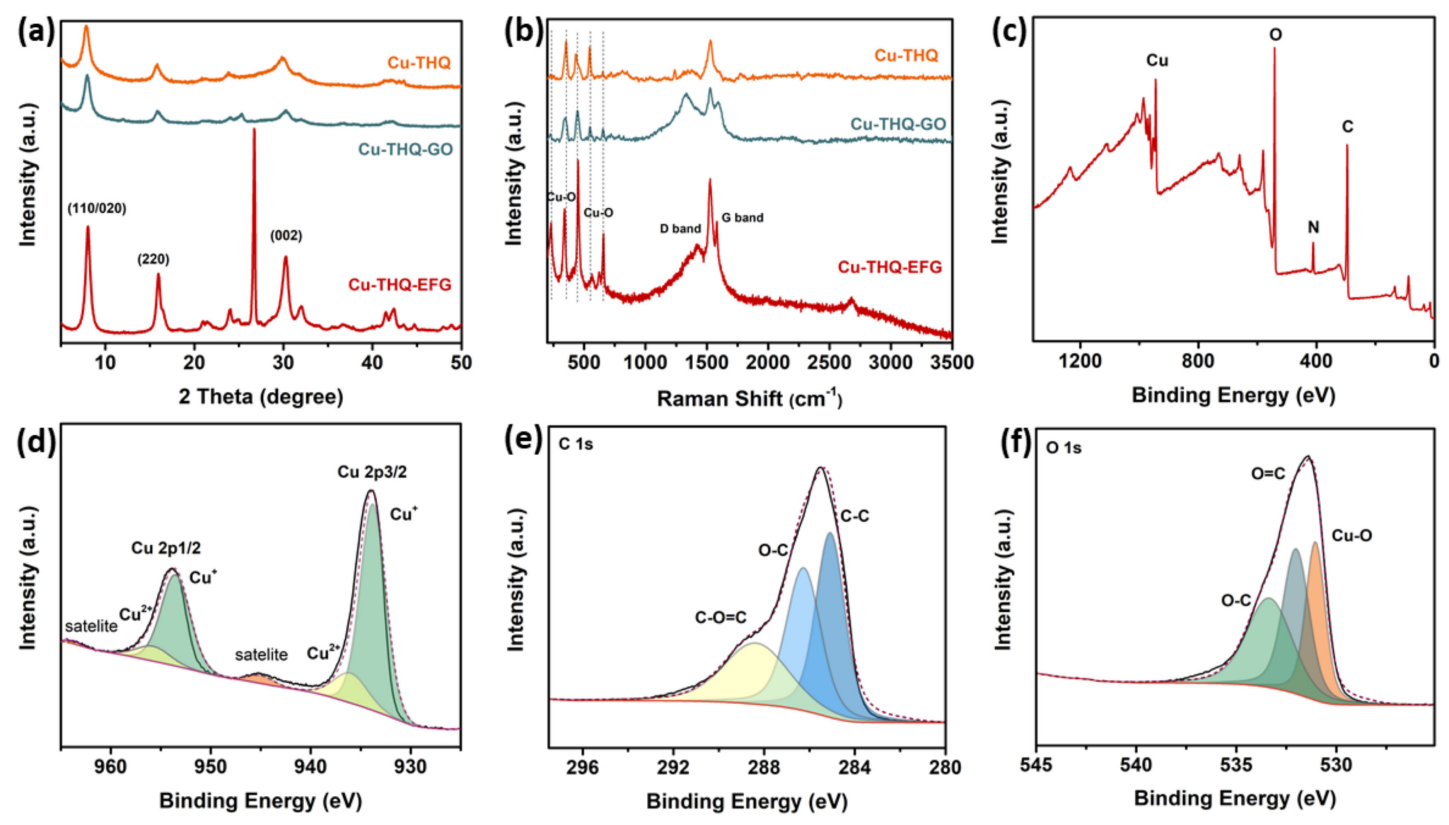
(**a**) XRD, (**b**) Raman spectra of pristine MOF (yellow curve), MOF/GO composite (green curve) and MOF/EFG composite (red curve); High resolution XPS spectra of (**c**) survey, (**d**) Cu 2p, (**e**) C 1s, (**f**) O 1s in the Cu-THQ-EFG.

The XRD data of Cu-THQ-EFG displays peaks at 8.0, 15.9 and 30.2°, corresponding to the planes of (100/020), (220) and (002), respectively, which matches well with the XRD patterns (Figure 2a) of pristine MOF and Cu-THQ-GO, moreover, they all agrees well with the XRD peaks reported in literature [28]. The strong peak at 27° belongs to the EFG substrate (Figure S5). This further demonstrates that MOF is successfully deposited on the EFG surface. Similarly, the weaken XRD peaks at around 10° and 30° of Cu-THQ and broaden peak of GO suggest that the Cu-THQ-GO composite was successfully synthesized (Figure S4). TGA curves of Cu-THQ and Cu-THQ-GO show the remaining weight percentage is about 38.3% after the temperature are higher than 400 °C (Figure S6a). The stable stage of Cu-THQ-EFG starts from as high as 650 °C, which demonstrates one of the benefits of EFG, that is, boosting the thermal stability of MOF composite compared to the GO substrate. The remaining weight percentage of Cu-THQ-EFG (30.8%) is insignificantly larger than that of Cu-THQ-GO (28.2%). Therefore, the loading mass of Cu-THQ-GO and Cu-THQ-EFG on carbon cloth as the working electrode are same as 0.83 mg/cm^2^, which can be calculated based on the TGA data. Raman analysis was employed to determine the vibrational modes of molecules. The spectra of pristine MOF, MOF on GO and on EFG are in good agreement with each other, except the signals of substrates. To be specific, the clear Raman responds at 245, 337, 455 and 658 cm^−1^ are associated with the spectrum of Cu-O bond in the three materials. Besides the two modes at 1330 cm^−1^ and 1587 cm^−1^ in the Raman spectra of EFG and GO (Figure S6b) correspond to disordered carbon (D band) and graphitic carbon (G band), respectively. As shown in the Raman modes of Cu-THQ-EFG and Cu-THQ-GO, they both obtain the D and G band, apparently gained from the substrate. This further suggests that the successful deposition of MOF on the two substrates took place.

XPS spectra of Cu-THQ-EFG validates the existence of Cu, C and O elements (Figure 2c) and therefore further suggests MOF deposition on the EFG surface. In Figure 2d, the binding characteristics of Cu 2p1/2 and Cu 2p3/2 at 933 and 952.8 eV clearly illustrate the Cu element in the catalyst, besides the two weak satellites and fitted two peaks in Cu spectrum shed light on the presence of Cu(II) and Cu(I) species, with Cu(I) being predominant. Furthermore, the binding energy of 288.5 eV in the C 1s spectrum (Figure 2e) clarifies the O-C=O coming from the substrate EFG with the edged carboxyl groups, which can be further proved by the C=O at binding energy of 533 eV in the O 1s spectrum (Figure 2f). Theoretically, the C=O bonds in Cu-THQ-EFG may also partially originate from the surplus THQ ligand. It also explains why the presence of C=O in the C 1 s XPS spectra of pristine MOF and Cu-THQ-GO as shown in Figures S7 and S8. For Cu-THQ and Cu-THQ-GO, the Cu 2p spectra also show the presence of Cu(II) and Cu(I) in the composite, indicating the chemical stability of Cu-THQ after growth on the substrate. The BET surface areas (S_BET_) of Cu-THQ-GO and Cu-THQ-EFG in Figure S9 was calculated to be 21.7 and 35.5 m^2^/g, respectively. The increase of S_BET_ value of the Cu-THQ-EFG benefits from the EFG substrate, which guarantees the MOF can be grown relatively uniformly on the substrate and decreases the aggregation of the bulk MOF.

### 3.2. CO_2_RR Performance Analysis

**Figure 3:**
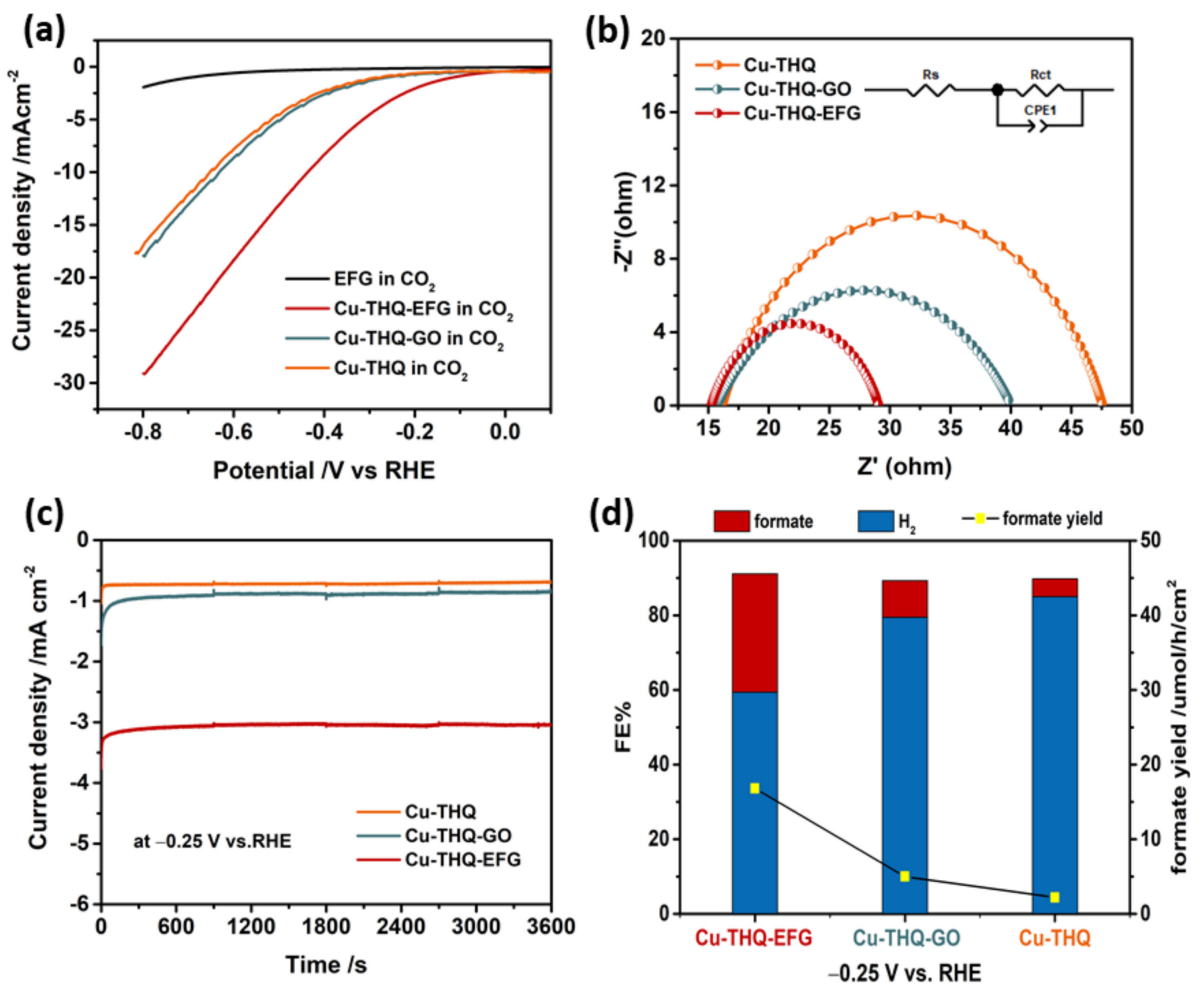
(**a**) LSV curves in 0.1 M NaHCO_3_ electrolyte, (**b**) EIS data of the Cu-THQ MOF, Cu-THQ-GO and Cu-THQ-EFG, respectively; (**c**) current density, (**d**) faradaic efficiency of H_2_ and formate yield for Cu-THQ, Cu-THQ-GO and Cu-THQ-EFG at the fixed potential −0.25 V vs. RHE.

The linear sweep voltammetry (LSV) in Figure 3a of Cu-THQ-EFG shows much larger current density at the same potential compared to the pristine Cu-THQ and Cu-THQ-GO. This result demonstrates that the number of transferred electron overcoming the barrier of electrolyte and electrode is increased by using EFG as substrate to improve the conductivity and mitigate the aggregation of the MOF. MOF/EFG composite has the on-set potential as −0.22 V vs. RHE, which is closer to the theoretical value of CO_2_/HCOOH reaction by 160 mV compared to the pristine MOF, of which the on-set potential has no significant difference with the one of Cu-THQ-GO (−0.38 V vs. RHE). EIS results (Figure 3b) show that using EFG in Cu-THQ-EFG composite reduces the catalytic resistance in CO_2_-saturated 0.1 M NaHCO_3_ aqueous solution. To be specific, the charge transfer resistance Rct of Cu-THQ-GO is 24 Ω, which is 7.4 Ω lower than Rct of pristine MOFs (31.4 Ω). Using EFG as a substrate, Rct decreases further to 13.8 Ω. Additionally, overall resistance (Rs) of Cu-THQ-EFG features a slightly lower value as 15.2 Ω compared to Cu-THQ-GO and pristine MOFs. This indicates Cu-THQ-EFG working as the electrode obtains a better interfacial contact of the electrolyte, compared with other two catalysts. Figure 3c shows the current density of three catalysts at −0.25 V vs. RHE and the highest current density at ~3 mA/cm^2^ is observed for Cu-THQ-EFG composite much larger than the current density of Cu-THQ-GO (0.87 mA/cm^2^) and Cu-THQ (0.72 mA/cm^2^). The three catalysts all exhibit stable performance at −0.25 V vs. RHE. In order to analyze the gas products from the process of electrolysis on Cu-THQ-EFG catalyst, the controlled potential electrolysis (CPE) experiments were performed at different fixed potentials from −0.1 to −0.5 V vs. RHE for 30 min (Figure S10). After process was running for 30 min, the produced gas mixture was injected into GC. According to the TCD data, only H_2_ detected (Figure S11a) and then quantified. On the other hand, formate as the liquid product in the electrolyte was detected by using NMR analysis. Figure S11b shows formate peak at 8.44 ppm and DSS peak at 0 ppm in ^1^H NMR data at the potential of −0.25 V vs. RHE. Applied several potentials from −0.1 V to −0.5 V vs. RHE to the Cu-THQ-EFG, the FE% of H_2_ and formate at each potential were calculated and shown in Figure S12a. Similarly, the results of FE% of H_2_ and formate produced by Cu-THQ-GO and Cu-THQ from −0.3 V to −0.6 V vs. RHE were exhibited in Figure S12b for comparison. It reveals that when Cu-THQ-GO is employed as the catalyst, the FE_formate_ reaches the value of 27% at potential of −0.5 V vs. RHE, which is larger than the FE_formate_ (less than 10%) of Cu-THQ. In fact, Cu-THQ-GO performs better selectivity for formate at the range of potential from −0.3 V to −0.6 V vs. RHE compared to the pristine MOF. Cu-THQ-EFG can produce more FE_formate_ of 31.7% at −0.25 V vs. RHE compared to Cu-THQ-GO (FE_formate_ = 9.8%) and Cu-THQ (FE_formate_ = 4.8%) (Figure 3d), which signifies MOF can compete with hydrogen evolution reaction (HER) in a relatively larger extent and achieve higher selectivity for formate. The black line in Figure 3d shows the yield of formate can achieve 16.8 μmol/h/cm^2^ at −0.25 V vs. RHE, which is 8 times than pristine MOF.

**Figure 4:**
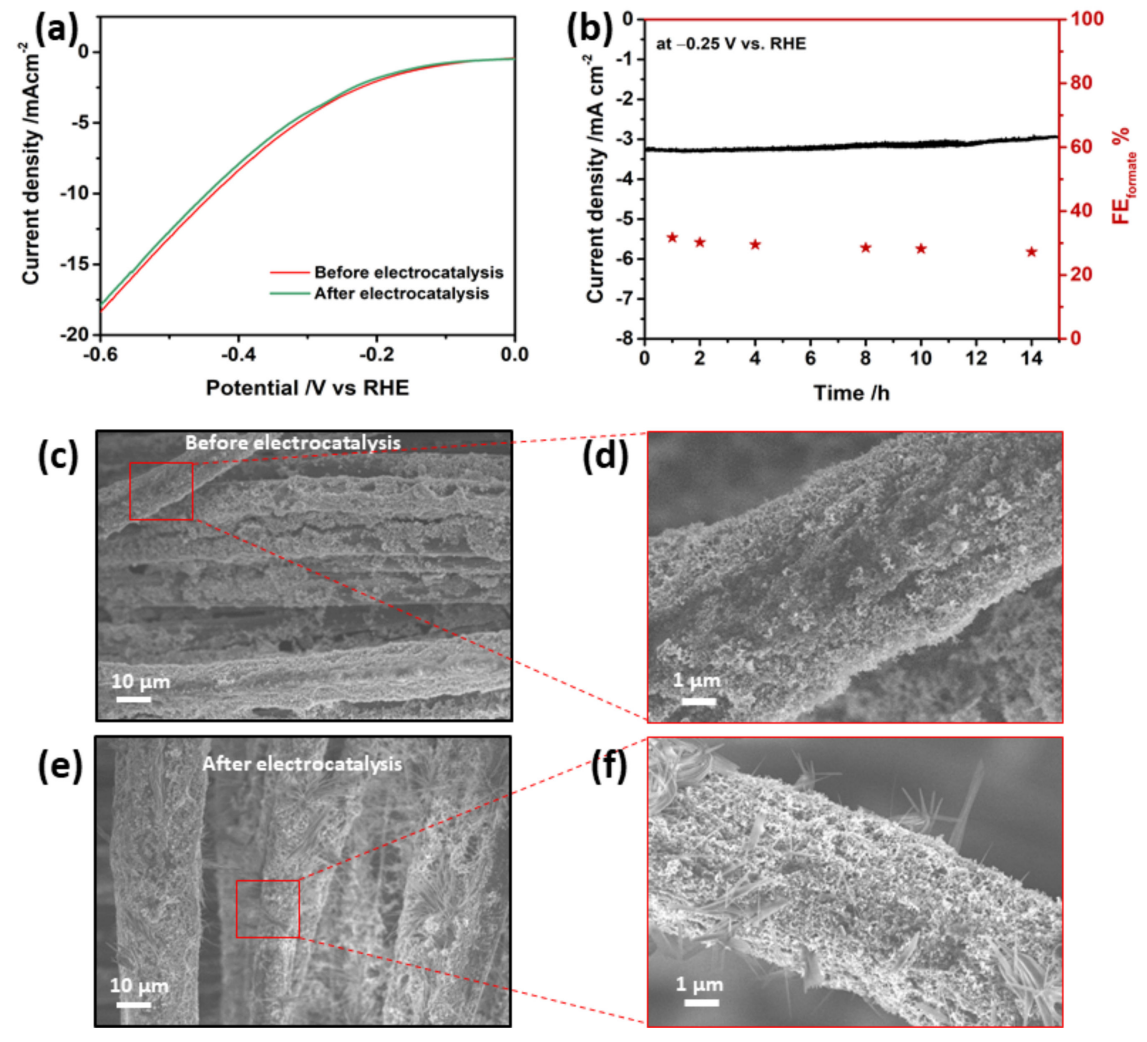
(**a**) LSV curves of Cu-THQ-EFG before and after electrocatalysis; (**b**) the long-term stability test of Cu-THQ-EFG at −0.25 V vs. RHE for 15 h; (**c**,**d**) SEM images of Cu-THQ-EFG before electrocatalysis; (**e**,**f**) SEM images of Cu-THQ-EFG after electrocatalysis.

**Scheme 2:**
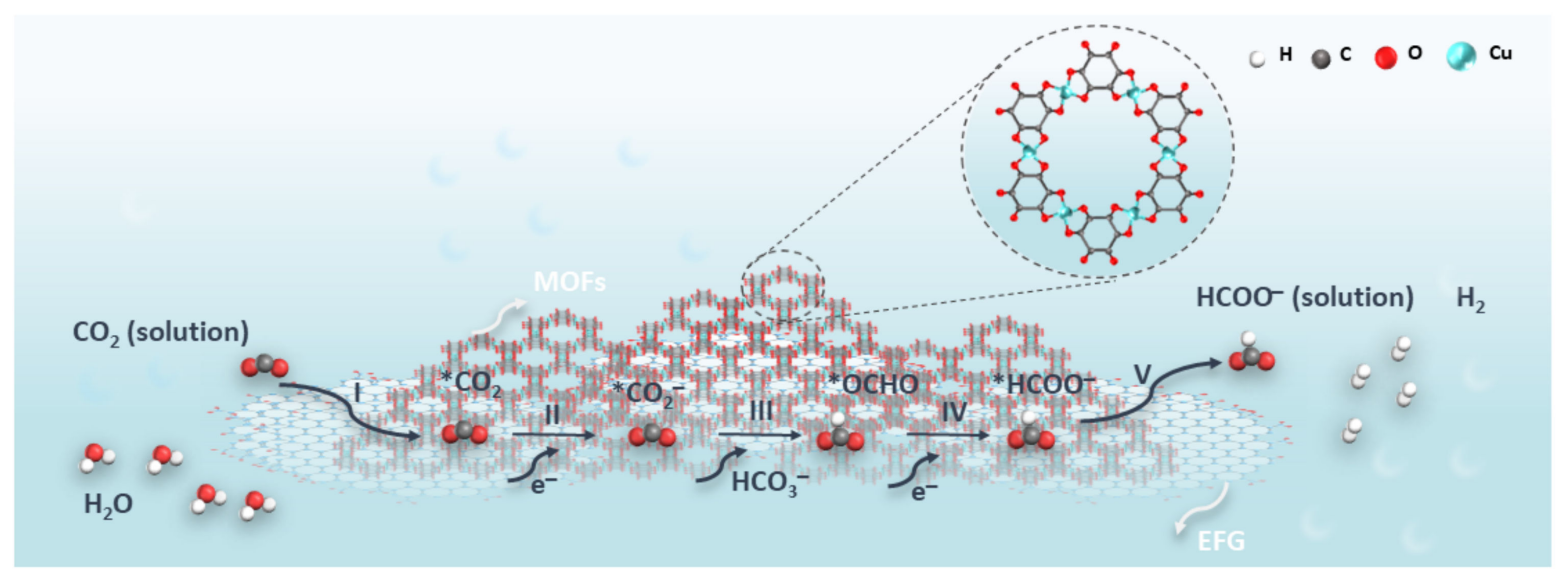
Illustration of the proposed electrocatalytic process of converting CO_2_ into formate.

Cu-THQ-EFG exhibits an excellent electrochemical stability as shown on Figure 4a. The LSV curves before and after electrolysis are overlapped well. The stability test of Cu-THQ-EFG was also conducted at −0.25 V vs. RHE and the result in Figure 4b shows the catalysis can be run continuously for at least 15 h with only slight drop in current density (0.22 mA/cm^2^). Additionally, the FE% of formate decreased from 31.7% to 28.2%, which further suggests the good catalytic stability of the MOF on EFG. The morphology of the working electrode before and after electrolysis have not significantly changed (Figure 4c–f). There are no more visual defects on the surface of carbon fiber after electrocatalysis, which further proves the good mechanical stability of the electrode. Scheme 2 illustrates the detailed electrocatalytic steps for formate generation. The step II is the rate determining step for the CO_2_RR, that is, transferring an electron to the absorbed CO_2_ (*CO_2_) (step I), followed by the protonation of *CO_2_ and conversion of *CO_2_ into the intermediate *OCHO (step III). Then, another electron is transferred and reduction of *OCHO into *HCOO take place (step IV). After the formate is released from the surface of the catalyst, the final step of the electrochemical reduction of CO_2_ is completed (step V).

## 4. Conclusions

In summary, we have fabricated the Cu-THQ-EFG composite by growing MOF onto EFG substrate. The morphology of Cu-THQ on EFG exhibits layered structure with less aggregation compared to the pristine MOF and MOF on GO. For electrocatalytic performance, Cu-THQ exclusively produces formate as the liquid product, which is easy to be purified from the aqueous electrolyte. The electrochemical results clarify the maximum FE_formate_ of 31.7% (yield of 16.8 μmol/h/cm^2^) can be achieved at potential only −0.25 V vs. RHE and the relevant current density can reach ~3 mA/cm^2^ in CO_2_-saturated 0.1 M NaHCO_3_. The selectivity for formate of Cu-THQ-EFG is higher than reported Cu-TCPP, Cu-SIM NU-1000 and other metal-based MOFs in the Table 1. The low potential and high selectivity for formate benefit from the higher conductivity and lower charge transfer resistance of Cu-THQ-EFG composite in CO_2_RR, compared to the pristine MOF and MOF-GO. Furthermore, the composite also exhibits excellent catalytic and mechanical stability at −0.25 V vs. RHE and can keep performing CO_2_RR activity for at least 15 h. Concluding, the addition of EFG as substrate into the MOF synthetic process is a general and scalable method. As thus, it can be extensively applied in synthesis of other MOF catalysts.

## Figures and Tables

**Table 1:** Comparison of the CO_2_RR performance with previously reported MOFs.

Catalyst	Electrolyte	Product	FE%	Potential (V vs. RHE)	Ref.
CR-MOF	0.5 M KHCO_3_	HCOOH	63	−0.76	[10]
Cu-SIM NU-1000	0.1 M NaClO_4_	HCOOH	28	−0.82	[12]
Cu_2_(CuTCPP) NSs	1 M H_2_O and 0.5 M EmimBF_4_	HCOOH	28.1	−1.4 V vs. Ag/Ag+	[13]
Cu-TCPP	0.5 M KHCO_3_	HCOOH	28.3	−0.7	[16]
Ag@Al-PMOF	0.1 M KHCO_3_	HCOOH	3.0	−1.1	[29]
HKUST-1	0.5 M KHCO_3_	C_2_H_5_OH/CH_3_OH	15.9	−0.37	[30]
RE-ndc-fcu-MOF@AuNMEs	0.1 M KHCO_3_	CH_4_	0.5 ± 0.1	−0.5	[31]
Zn-MOF-74	0.5 M KHCO_3_	CO	21.72	−0.91	[32]
ZIF-8	0.5 M NaHCO_3_	CO	65	−1.62	[33]
Cu_3_(BTC)_2_	0.01 M MTBAEFB/DMF	C_2_H_2_O_4_	51	−1.12 V vs. Ag/AgCl	[34]
CuAdeAce	0.5 M KHCO_3_	CH_3_OH/C_2_H_5_OH	1.2	−1.75	[35]
Cu-THQ-EFG	0.1 M NaHCO_3_	HCOOH	31.7	−0.25	This work

## Data Availability

Not Applicable.

## Supplementary Materials

The following supporting information can be downloaded at: https://www.mdpi.com/article/10.3390/polym14235112/s1, Figure S1: Digital image of the on-line gas analysis system employed for CO_2_ electrocatalysis; insert is an enlarged image of the H-cell with two compartment; Figure S2: Calibration curve of gas product, (a) CH_4_, (b) CO and (c) H_2_ and liquid product, (d) HCOOH in electrocatalysis; Figure S3: SEM images of Cu-THQ-GO composite; Figure S4: TEM image of Cu-THQ-EFG composite; (b) enlarged TEM iamge of Cu-THQ phases in the composite; Figure S5: XRD curves of two substrate (GO and EFG); Figure S6: (a)TGA curves of two substrate (GO and EFG) and three catalysts (Cu-THQ, Cu-THQ-GO and Cu-THQ-EFG), (b) Raman spectrums of two substrate (GO and EFG); Figure S7: XPS of Cu-THQ; Figure S8: XPS of Cu-THQ-GO; Figure S9: N_2_ sorption isotherms of Cu-THQ-EFG composite; Figure S10: Controlled potential electrolysis (CPE) measurements at fixed potentials from −0.1 V to −0.5 V vs. RHE; Figure S11: (a) GC and (b) NMR data of Cu-THQ-EFG at −0.25 V vs. RHE; Figure S12: (a) Faradaic efficiency of H_2_ and formate for Cu-THQ-EFG at fixed potentials from −0.1 V to −0.5 V vs. RHE; (b) Faradaic efficiency of H_2_ and formate for Cu-THQ and Cu-THQ-GO at fixed potentials from −0.3 V to −0.6 V vs. RHE.
