# Supplementary material for: Cu-THQ-EFG Composite for Highly Selective Electrochemical CO2 Reduction to Formate at Low Overpotentials
Source: Polymers (Basel). 2022 Nov 24;14(23):5112. doi: 10.3390/polym14235112 (PMC9737261; doi:10.3390/polym14235112)
Supplement: Supplementary file 1 [file polymers-14-05112-s001.zip › polymers-2013684-supplementary.pdf]

polymers

Supplementary

Cu-THQ-EFG Composite for Highly Selective Electrochemical CO_2 Reduction to Formate at Low Overpotentials

Lisha Jia, Klaudia Wagner, Jamie Smyth, David Officer, Jun Chen * and Pawel Wagner *

ARC Centre of Excellence for Electromaterials Science, Intelligent Polymer Research Institute, University of Wollongong, Wollongong, NSW 2522, Australia

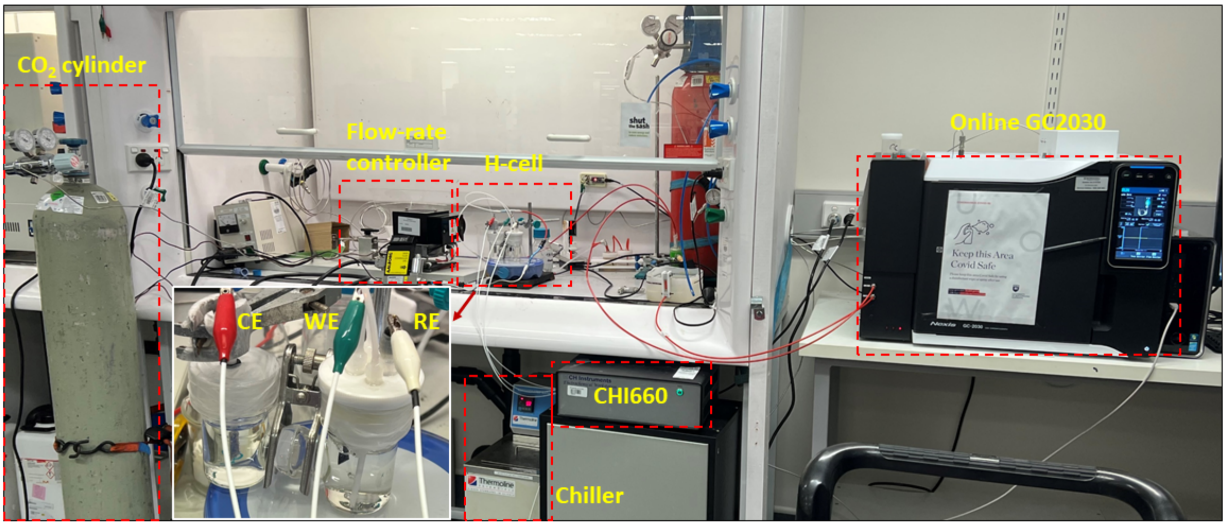

Figure S1. Digital image of the on-line gas analysis system employed for CO_2 electrocatalysis; insert is an enlarged image of the H-cell with two compartment.

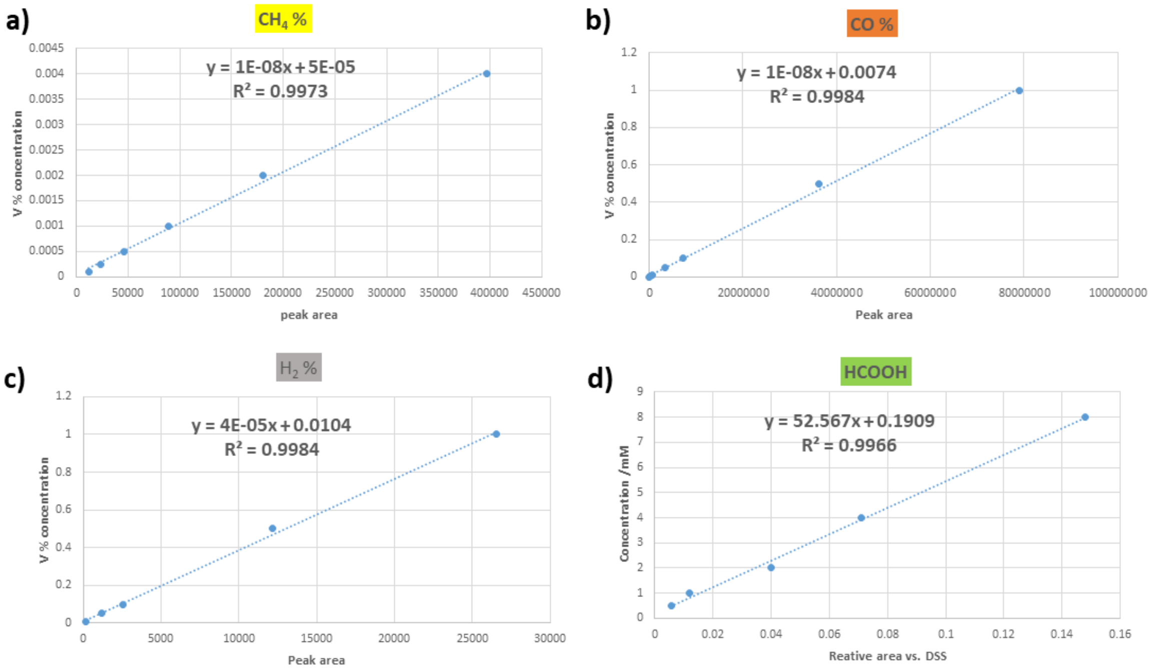

Figure S2. Calibration curve of gas product, a) CH_4, b) CO and c) H_2 and liquid product, d) HCOOH in electrocatalysis.

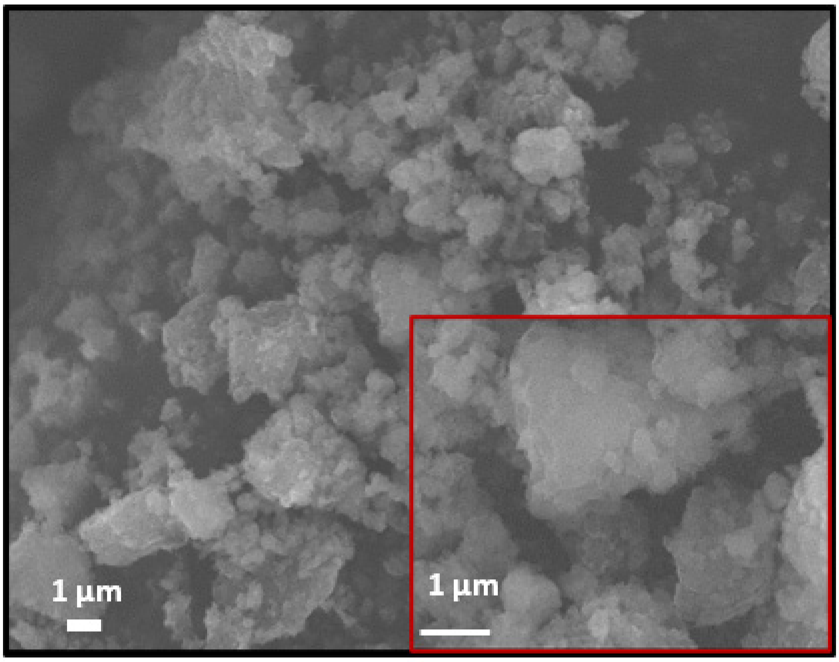

Figure S3. SEM images of Cu-THQ-GO composite.

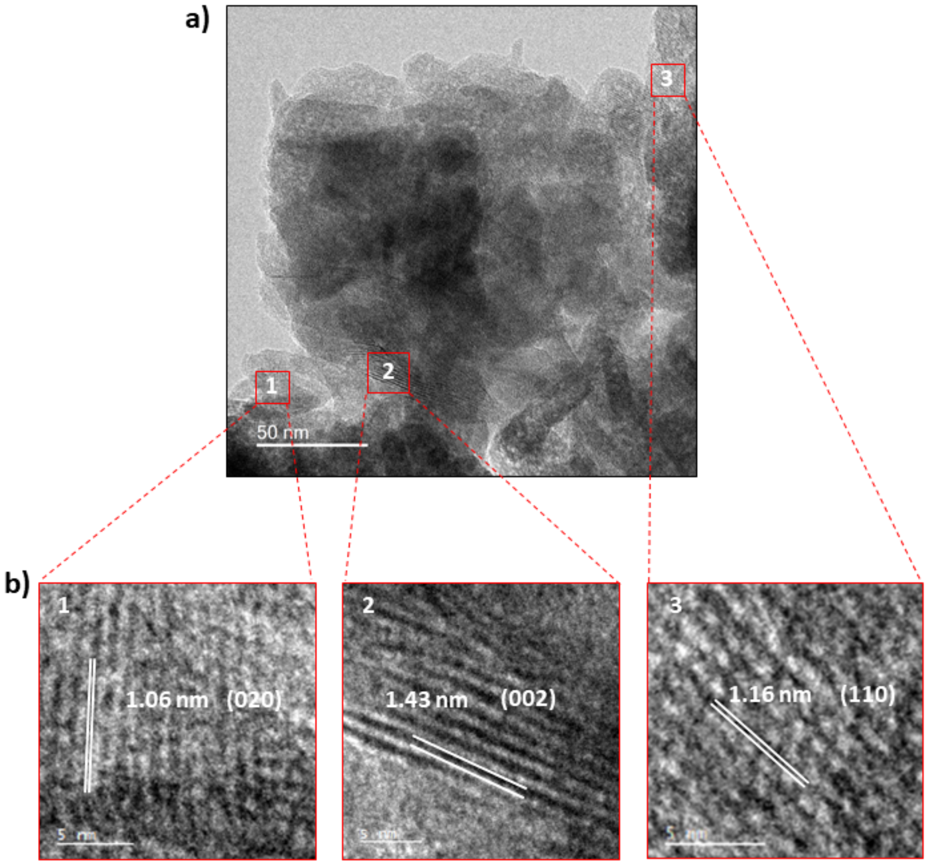

Figure S4. a) TEM image of Cu-THQ-EFG composite; b) enlarged TEM image of Cu-THQ phases in the composite.

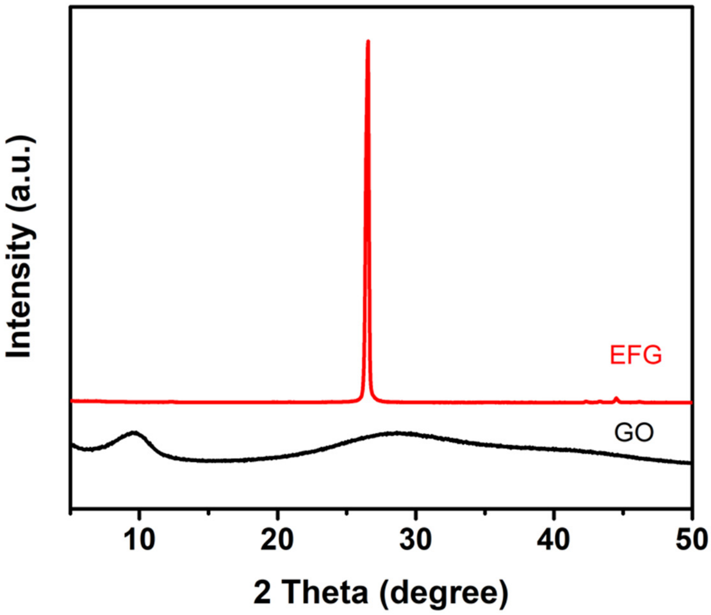

Figure S5. XRD curves of two substrate (GO and EFG).

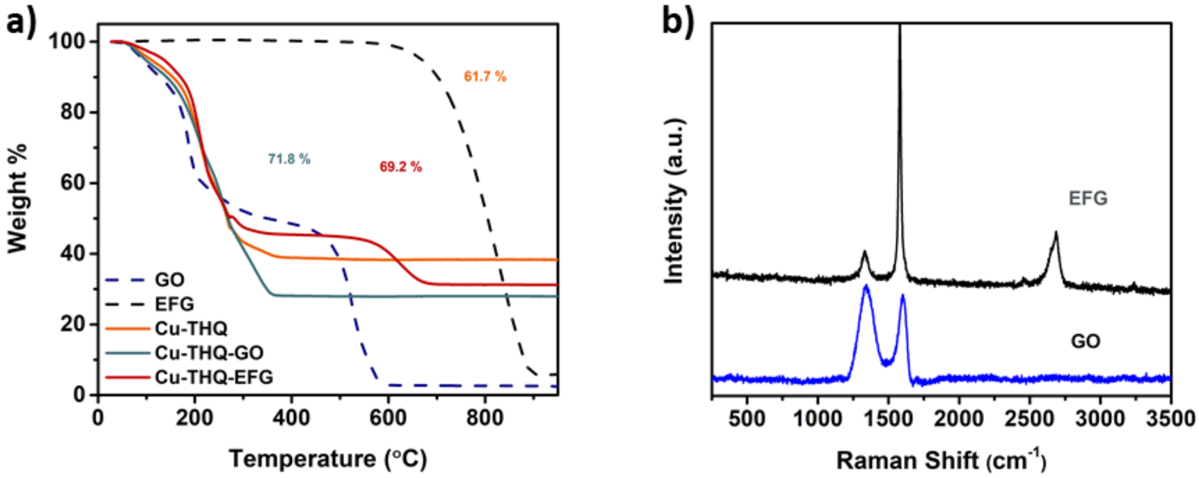

Figure S6. a)TGA curves of two substrate (GO and EFG) and three catalysts (Cu-THQ, Cu-THQ-GO and Cu-THQ-EFG), b) Raman spectrums of two substrate (GO and EFG).

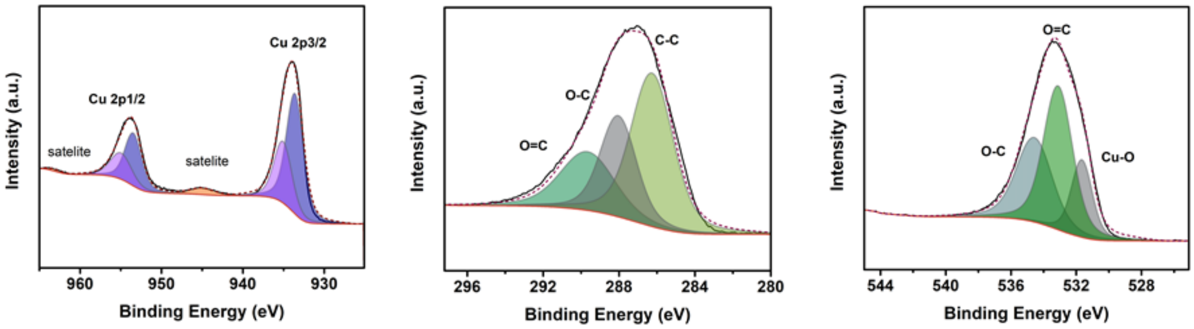

Figure S7. XPS of Cu-THQ.

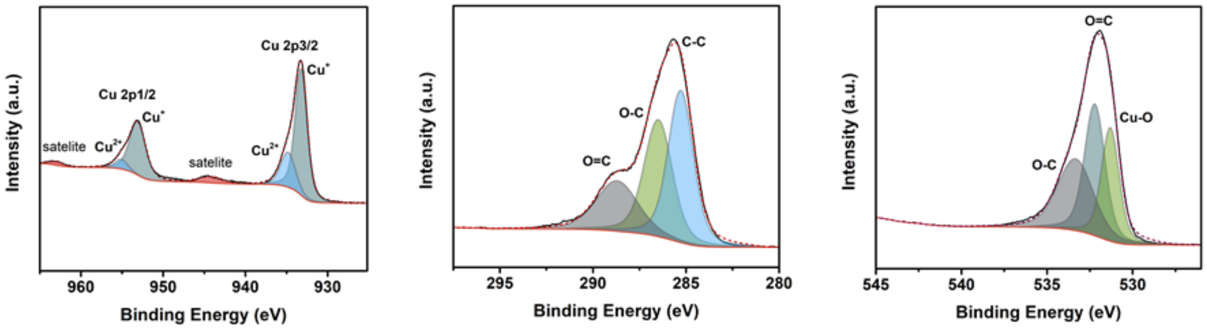

Figure S8. XPS of Cu-THQ-GO.

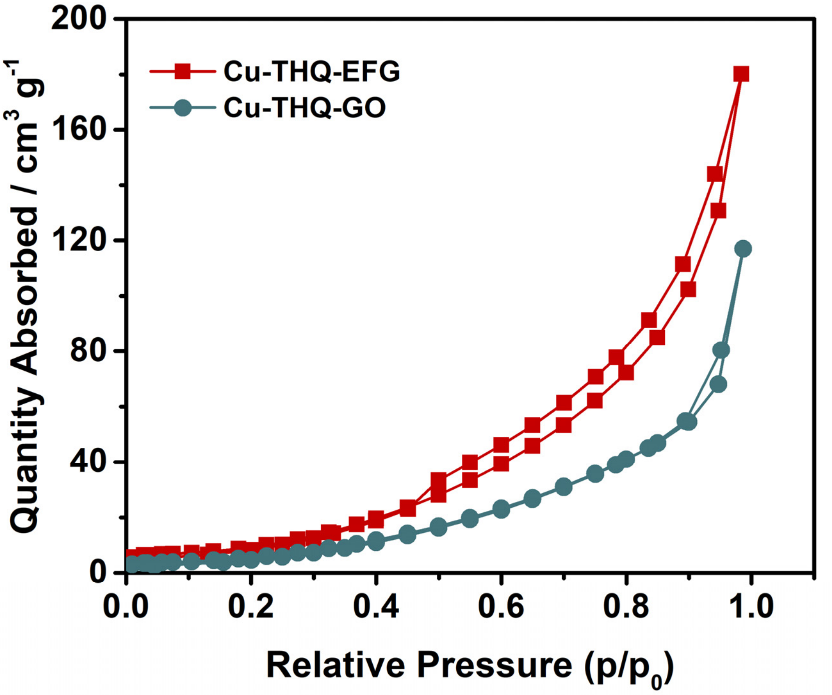

Figure S9. N_2 sorption isotherms of Cu-THQ-GO and Cu-THQ-EFG composite.

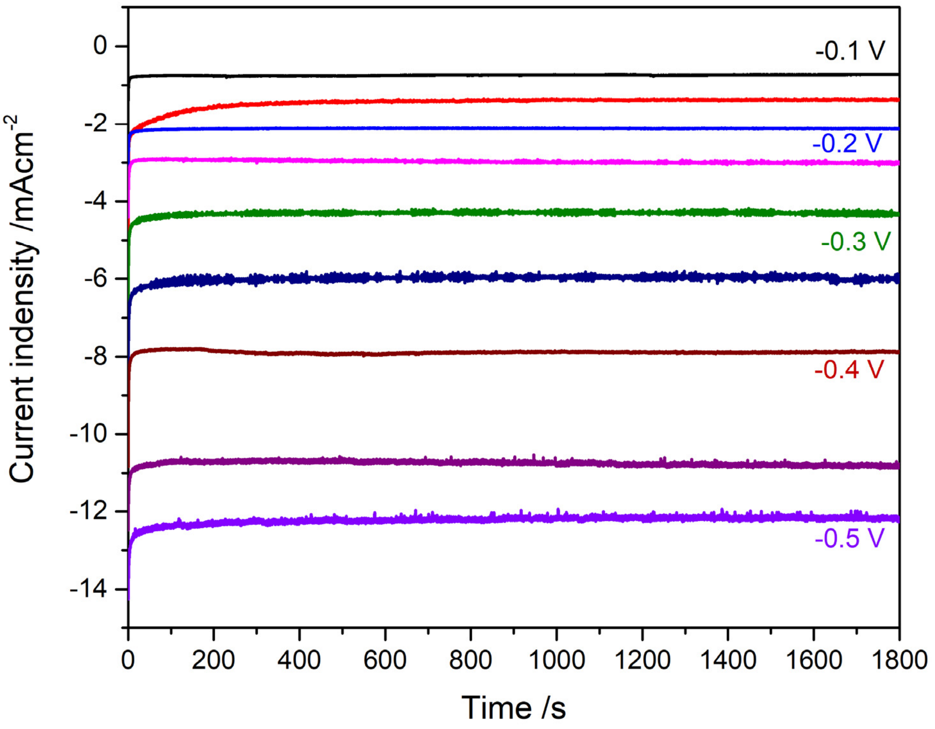

Figure S10. Controlled potential electrolysis (CPE) measurements at fixed potentials from -0.1 V to -0.5 V vs. RHE.

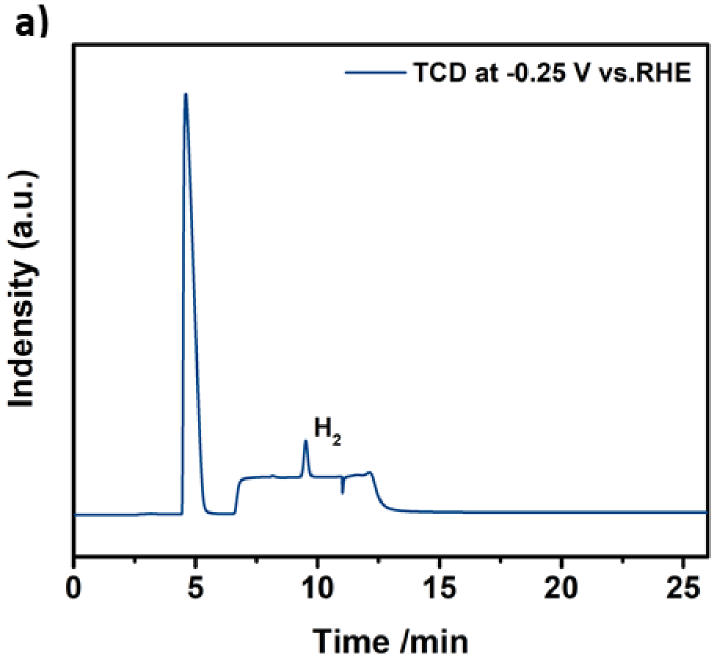

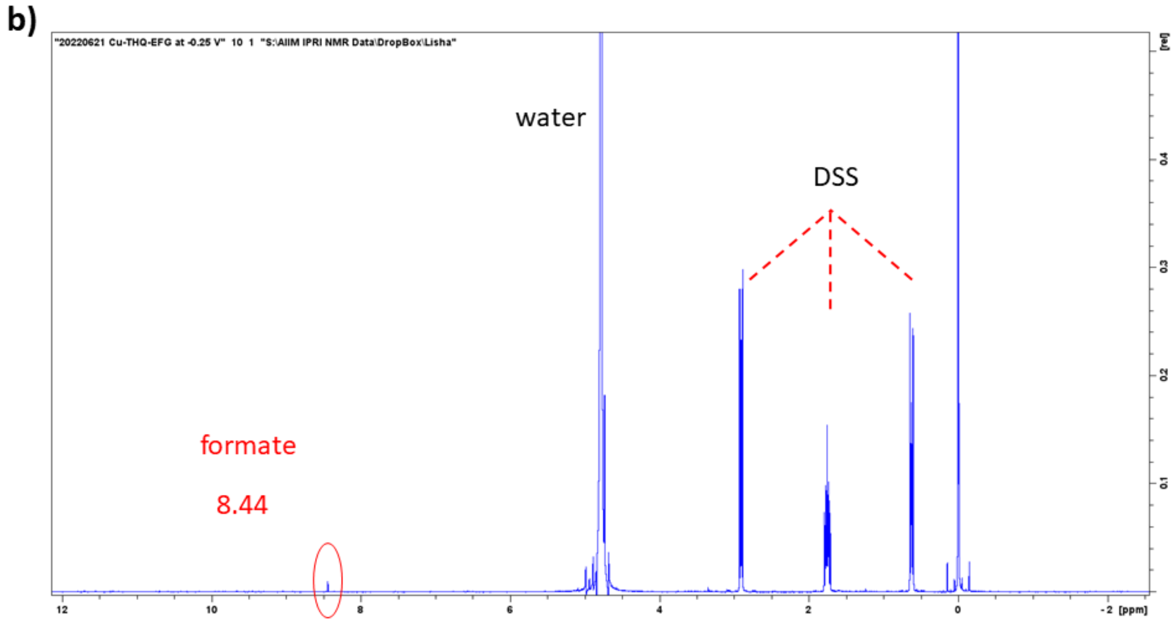

Figure S11. a) GC and b) NMR data of Cu-THQ-EFG at -0.25 V vs. RHE.

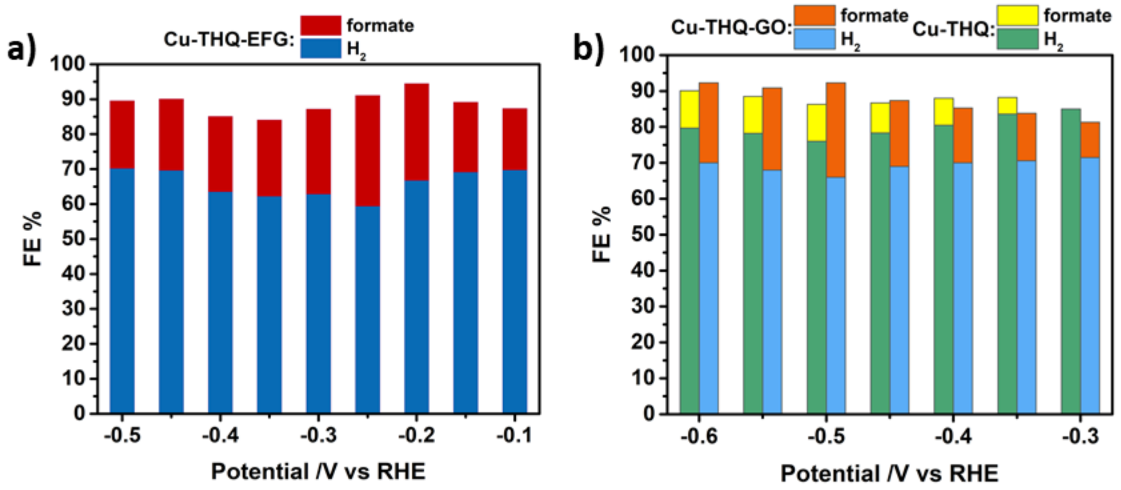

Figure S12. a) Faradaic efficiency of H_2 and formate for Cu-THQ-EFG at fixed potentials from -0.1 V to -0.5 V vs RHE; b) Faradaic efficiency of H_2 and formate for Cu-THQ and Cu-THQ-GO at fixed potentials from -0.3 V to -0.6 V vs. RHE.
